# Executive functioning in preschoolers with specific language impairment

**DOI:** 10.3389/fpsyg.2015.01574

**Published:** 2015-10-20

**Authors:** Constance Vissers, Sophieke Koolen, Daan Hermans, Annette Scheper, Harry Knoors

**Affiliations:** ^1^Kentalis Academy, Royal Dutch KentalisSint-Michielsgestel, Netherlands; ^2^Behavioural Science Institute, Radboud UniversityNijmegen, Netherlands; ^3^Vincent van Gogh for PsychiatryVenray, Netherlands

**Keywords:** specific language impairment, preschoolers, executive functioning, working memory capacity, inhibition (psychology), shifting

## Abstract

The pathogenesis of Specific Language Impairment (SLI) is still largely beyond our understanding. In this review, a neuropsychological perspective on language impairments in SLI is taken, focusing specifically on executive functioning (EF) in preschoolers (age range: 2.6–6.1 years) with SLI. Based on the studies described in this review, it can be concluded that similar to school-aged children with SLI, preschoolers with SLI show difficulties in working memory, inhibition and shifting, as revealed by both performance based measures and behavioral ratings. It seems plausible that a complex, reciprocal relationship exists between language and EF throughout development. Future research is needed to examine if, and if yes how, language and EF interact in SLI. Broad neuropsychological assessment in which both language and EF are taken into account may contribute to early detection of SLI. This in turn can lead to early and tailored treatment of children with (suspected) SLI aimed not only at stimulating language development but also at strengthening EF.

## Introduction

Children with an unexplained severe delay in the development of speech and language are described as having Specific Language Impairment (SLI). SLI is determined by applying exclusionary criteria; hence defined by what it is not rather than by what it is. Children are diagnosed with SLI when exhibiting significant language disabilities which cannot be explained in terms of a sensory deficit, neurological disorder, intellectual impairment, psychiatric diagnosis, or a lack of exposure to language (Bishop, 1997). In SLI, language difficulties are present from the outset of the language-learning process (Conti-Ramsden and Durkin, 2012). The prevalence of children with SLI varies from 5 to 10% in the population (Law et al., 1998). SLI is a heterogeneous classification. Variation exists, both in the aspects of language that are affected and in the severity in which these linguistic deficits are found (Aram and Nation, 1975; van Weerdenburg et al., 2006). At classification level, SLI co-occurs with other developmental disorders like autism spectrum disorder (ASD; e.g., Conti-Ramsden et al., 2006) and attention-deficit/hyperactivity disorder (ADHD; e.g., Cohen et al., 1998).

The pathogenesis of SLI is still largely beyond our understanding. Both etiology of SLI and neurobiological contributions are not yet clearly understood (Verhoeven and van Balkom, 2004; Bishop, 2006). Etiologically, genetic mutations have been identified in a small number of cases of SLI, overall though genetic variants in SLI seem the same as in the typical population (Fisher and Scharff, 2009; Kang and Drayna, 2011; Simpson et al., 2015). At a neurobiological level, it is stated that children with language impairments have atypical brain structure and function within neural regions integral to language (Mayes et al., 2015). Genetics and neurobiology cannot explain the causes of SLI on its own. After all, genes do not cause language impairments in isolation and abnormal brain structure and function can be both cause and consequence of language impairments. Neuropsychology provides a framework for differentiating language impairments in SLI in terms of interaction between genes, brain, cognition, experiences, and context. In this review, a neuropsychological perspective on language impairments in SLI is taken, focusing specifically on executive functioning (EF) in preschoolers with SLI. Herewith, we aim to contribute to understanding the pathogenesis of SLI and to tailored neuropsychological assessment and treatment.

When cognitively differentiating language and behavioral problems in children with developmental disorders, impairments in EF are often brought to light (e.g., Hill, 2004; Bishop and Norbury, 2005; Castellanos et al., 2006). EF can be defined as the top-down control of cognitive processes for goal achievement; EFs are necessary in the regulation of more automatic processes (thoughts, behavior, emotion) in the service of a goal (Miyake et al., 2000). A well-established conceptualization of EF is Miyake's model, which proposes EF as a unitary construct with three separable components; inhibition of pre-potent responses, mental set shifting, and information updating/monitoring of working memory (WM) representations (see Table 1 for an overview of EF components and related tasks). In conceptualizing WM, Baddeley's multicomponent WM model is mostly used (Baddeley, 2000). According to this model, a central executive system (CE) is proposed to be linked to three subsystems: the phonological loop, the visuo-spatial sketchpad, and the episodic buffer. The CE is responsible for attentional control. The phonological loop and visuo-spatial sketchpad are “slave”-systems and are responsible for temporary storing information. The episodic buffer is proposed to integrate representations from WM, long-term memory and language processing systems.

**Table 1:** **Overview of Miyake's three EF components (Updating/WM, Inhibition and Shifting), and related EF tasks**.

**EF component**	**Task**	**Task description**
**UPDATING/WORKING MEMORY**		
The ability to (temporarily) store and monitor incoming information and then update items in WM with new, more relevant information.	Digit recall (Petrucelli et al., 2012)	Subtest from the Working Memory Test Battery for Children measuring phonological WM, in which numbers have to be repeated.
	Children's Test of Non-word Repetition (Petrucelli et al., 2012)	A test in which unfamiliar words spoken by an experimenter have to be repeated, measuring phonological WM.
	Recalling Sentences Task (Petrucelli et al., 2012)	Subtest from the Clinical Evaluation of Language Fundamentals in which sentences have to be imitated, measuring the episodic buffer^a^.
	Non-word repetition (Chiat and Roy, 2007)	Part of the Preschool Repetition Test, in which words and phonologically matched non-words have to be repeated, measuring verbal WM.
	Non-word repetition (Gray, 2006)	A test in which lists of spoken words and non-words (one-, two-, three-, four- syllable non-words) have to be repeated, measuring verbal WM.
	Digit span (Gray, 2006)	A test in which a sequence of digits (varying from three to nine numbers in length) has to be recalled in the right order, measuring verbal WM storage.
	Digit recall task (Vugs et al., 2014, 2015)	Subtest of the Automated Working Memory Assessment, in which a sequence of digits has to be recalled in the right order, measuring verbal WM storage.
	Word recall task (Vugs et al., 2014, 2015)	Subtest of the Automated Working Memory Assessment, in which a sequence of words has to be recalled in the right order, measuring verbal WM storage.
	Non-word recall task (Vugs et al., 2014)	Subtest of the Automated Working Memory Assessment, in which a sequence of non-words has to be recalled in the right order, measuring verbal WM storage.
	Listening span task (Vugs et al., 2014, 2015)	Subtest of the Automated Working Memory Assessment, in which the content of presented sentences has to be judged while the last word of the sentence has to be remembered. Afterwards, the last words of the sentences have to recalled in the correct order. A measure for the verbal central executive.
	Counting recall task (Vugs et al., 2014, 2015)	Subtest of the Automated Working Memory Assessment, in which dots (presented amongst triangles) have to be counted, said out loud and remembered. Afterwards, the number of dots have to be recalled in the correct order. A measure for the verbal central executive.
	Backward digit recall task (Vugs et al., 2014, 2015)	Subtest of the Automated Working Memory Assessment, in which a sequence of digits has to be recalled in the reversed orders, measuring the verbal central executive.
	Dot matrix task (Vugs et al., 2014, 2015)	Subtest of the Automated Working Memory Assessment, in which a sequence of dots is presented and then disappears, after which the position of the dots has to be pointed out in the correct serial order. A measure for visuo-spatial storage.
	Mazes memory task (Vugs et al., 2014, 2015)	Subtest of the Automated Working Memory Assessment, in which a maze with a path drawn through it is presented for 3 s, after which the path has to be drawn in a similar but “empty” maze. A measure for visuo-spatial storage.
	Block recall task (Vugs et al., 2014, 2015)	Subtest of the Automated Working Memory Assessment, in which nine cubes are presented and then pointed to in a particular order. Afterwards, the cubes have to be pointed to in the correct order. A measure for visuo-spatial storage.
	Odd-one-out task (Vugs et al., 2014, 2015)	Subtest of the Automated Working Memory Assessment, in which three boxes with complex shapes are presented. The shape that does not resemble the others has to be identified. After a number of trials, the position of the boxes containing the odd shapes has to be recalled in the correct order. A measure for the visuo-spatial central executive.
	Mr. X task (Vugs et al., 2014, 2015)	Subtest of the Automated Working Memory Assessment, in which the position of balls held by one of two men has to be judged. Afterwards the position of the balls has to be recalled. A measure for the visuo-spatial central executive.
	Spatial span task (Vugs et al., 2014, 2015)	Subtest of the Automated Working Memory Assessment, in which the similarity of shapes has to be judged. A red dot is presented above the right shapes. Afterwards, the position of the red dots has to be recalled in the right order. A measure for the visuo-spatial central executive.
	Word span (Hick et al., 2005)	A test in which lists of spoken words of increasing length (two, three, four or five words) have to be repeated, measuring verbal WM.
	Pattern recognition memory (Bavin et al., 2005)	A visuo-spatial short-term memory task, in which a series of patterns appear on a screen. Afterwards, two patterns are presented: a new one and one of the previously presented patterns. The pattern that was presented before has to be selected.
	Paired associates learning (Bavin et al., 2005)	A visuo-spatial short-term memory task, in which boxes are presented, opening and closing one at a time. One of the boxes contains a target item. Afterwards the boxes appear again, now in the middle of the screen. Then the box in which the target appeared before has to be selected.
	Localization recall task (Menezes et al., 2007)	A visual short-term memory task, in which two boxes are presented on the table, in which target objects are put. The task is to identify and retrieve the target items (distractor objects are also presented).
	Space Visualization Task (Marton, 2008)	A task in which wooden blocks have to be mentally rotated to fit pegs into various holes. One of two alternatives has to be chosen. This task measures, amongst other executive skills, visuo-spatial WM.
	Position in Space Task (Marton, 2008)	The task is first to match a series of figures to visually similar abstract forms, and then to remember a row of figures that was previously presented. This task measures, amongst other executive skills, visuo-spatial short-term storage.
	Design Copying Task (Marton, 2008)	The task is to copy lines and abstract figures in empty spaces. This task measures, amongst other executive skills, visuo-spatial WM.
	Working memory scale BRIEF-P (Vugs et al., 2014)	The BRIEF-P is a standardized rating scale for parents and teachers measuring EF behaviors of children aged 2–5 years. The scale contains 63 items divided across five clinical scales, including a WM scale.
	Emergent Metacognition Index BRIEF-P (Wittke et al., 2013)	The BRIEF-P is a standardized rating scale for parents and teachers for measuring EF behaviors of children aged 2–5 years. The scale contains 63 items divided across five clinical scales. The scales form a Global Executive Composite, and three overlapping summary indices. One of these indices is the Emergent Metacognition Index, composed of the WM and the planning/organization scales.
**INHIBITION**		
The ability to deliberately inhibit dominant or automatic responses and to resistance to distractor interference (Friedman and Miyake, 2004).	Resistance to distractor interference task (Spaulding, 2010)	A task using speech, environmental sounds, and visual animations. The task is to press a button when a target item is named by a speaker, while visual, non-verbal, and linguistic distractors are presented that need to be resisted.
	Inhibition task (Spaulding, 2010)	A task based on a stop-signal paradigm, using linguistic recordings. The task is to press the button with a picture of a butterfly when presented with this word, or a button with a picture of a dinosaur when presented with that word. When the spoken word was followed by the word “stop,” the response needed to be inhibited.
	Inhibition scale BRIEF-P (Vugs et al., 2014)	The BRIEF-P is a standardized rating scale for parents and teachers for measuring EF behaviors of children aged 2–5 years. The scale contains 63 items divided across five clinical scales, including an inhibition scale.
	Inhibitory Self-Control Index BRIEF-P (Wittke et al., 2013)	The BRIEF-P is a standardized rating scale for parents and teachers for measuring EF behaviors of children aged 2–5 years. The scale contains 63 items divided across five clinical scales. The scales form a Global Executive Composite, and three overlapping summary indices. One of these indices is the Inhibitory Self-Control Index, composed of the inhibition and emotional self-control scales.
**SHIFTING**		
The ability to disengage from a task set and to actively engage in a new task set. The ability to shift is strongly related to cognitive flexibility.	Flexible Item Selection Task (Roello et al., 2015)	A task measuring categorization and shifting abilities. The task is first to select pictures matching for one feature, than to choose a different pair of pictures matching for another feature.
	Border version of the Dimensional Change Card Sort (Farrant and Maybery, 2012)	The task is to sort a series of bivalent multidimensional cards. In the pre-switch phase the cards are sorted along one dimension, in the post-switch phase the cards are sorted along another dimension, and in the border phase the cards are sorted along both dimensions depending upon whether the card has a border or not.
	Shifting scale BRIEF-P (Vugs et al., 2014)	The BRIEF-P is a standardized rating scale for parents and teachers for measuring executive function behaviors of children aged 2–5 years. The scale contains 63 items divided across five clinical scales, including a shifting scale.
	Flexibility Index BRIEF-P (Wittke et al., 2013)	The BRIEF-P is a standardized rating scale for parents and teachers for measuring executive function behaviors of children aged 2–5 years. The scale contains 63 items divided across five clinical scales. The scales form a Global Executive Composite, and three overlapping summary indices. One of these indices is the Flexibility Index, composed of the shifting and emotional self-control scales.

*Exclusively those tasks are presented on which preschoolers with SLI perform significantly worse than their typically developing peers*.

a *Note that some authors take sentence-recall tasks as a measure of the episodic buffer (e.g., Petrucelli et al., 2012), while other take it as a measure of language ability (e.g., Klem et al., 2015)*.

It is well-established that EFs are closely related to language in typically developing children (e.g., Carlson et al., 2005). Lining up with this, school-aged children with SLI are found to suffer from impairments on both WM, inhibition, and shifting. Specifically, studies have found limitations on phonological WM tasks in schoolchildren with SLI (Marton and Schwartz, 2003; Archibald and Gathercole, 2006; Bishop, 2006; Im-Bolter et al., 2006; Montgomery et al., 2010; Duinmeijer et al., 2012; Henry et al., 2012; Conti-Ramsden et al., 2015). Although, some studies show that children with SLI perform similar to their typically developing peers on visuo-spatial WM tasks (e.g., Lum et al., 2012), a meta-analysis suggests that WM deficits do extend to the visuo-spatial domain (Vugs et al., 2013). With regard to inhibition processes, children with SLI are shown to have difficulties inhibiting pre-potent responses (e.g., Bishop and Norbury, 2005; Marton et al., 2007) and to be more susceptible to distraction (Lum and Bavin, 2007). With respect to cognitive flexibility some studies do not show deficits in children with SLI (e.g., Kiernan et al., 1997; Im-Bolter et al., 2006), while others reveal attentional shifting problems and cognitive inflexibility (Marton, 2008; Henry et al., 2012).

All of these studies were performed on school-aged children. Only little is known about EF in preschool children with SLI. Studies in typically developing preschoolers show that important progress in EF is made during this period. Evidence for structural changes within the prefrontal cortex during preschool period—a brain region that is established to show an important role in EF—lines up to this dramatic progress in EFs (Moriguchi and Hiraki, 2013). EF is proposed to develop in a hierarchical matter, with attention serving as the foundation: simpler EF components (e.g., behavioral inhibition) develop during the first 3 years in life, and then become integrated into more complex EF processes (e.g., planning) (Garon et al., 2008). Both the development of WM and shifting starts during preschool (e.g., Huizinga et al., 2006; Best and Miller, 2010). The ability to inhibit pre-potent responses also increases dramatically in preschool years (Jones et al., 2003). Furthermore, growth in resistance to distractor interference occurs during preschool period, but it develops at a slower rate and continues to develop until pre-teen years (Bjorklund and Harnishfeger, 1990; Ruff and Capozzoli, 2003). Complex EF abilities that develop later, are said to be constructed from earlier developed EF abilities. Since early childhood is the primary period for both language and EF to develop, the early development of language and EF plausibly interact in an empowering or inhibitive manner. To come to early detection, to tailored treatment and ultimately to insight into the pathogenesis of SLI, research on the construct and measurement of early EF and language development and their existing deficits is necessary.

In the next part of this review state of the art evidence on EF of preschoolers with SLI (age range: 2.6–6.1 years) is presented. Next to performance-based tasks of EF, rating scales of everyday EF behavior in home and school settings are reviewed. We end with an elaboration of theoretical and clinical implications of these empirical data and suggestions for future research.

## Executive functioning in preschoolers with SLI

### Updating and working memory

Until now, only few studies have examined WM profiles of preschoolers with SLI.

Findings from a study by Petrucelli et al. (2012) suggest that, preschoolers with SLI have limited phonological WM capacity, as evidenced by poor performance on a digit recall task and a non-word repetition task. In this study, young children with SLI also showed problems with regard to the episodic buffer, as tapped by poor performance on a sentence-recall task. With respect to visual-spatial WM and the central executive, no differences between SLI and typical children were found. Chiat and Roy (2007) also found a verbal WM deficit as measured by a non-word repetition task in a clinical group of preschool children, who were referred to speech and language therapy. The oldest clinical group (3–4 years) showed a performance profile close to the youngest typical group, which was on average 18 months younger. Gray (2006) assessed phonological WM in preschoolers using a non-word repetition task and digit span task. This study revealed that phonological memory skills of preschoolers with normal language and SLI increased between ages 3 and 4 and remained relatively stable from 4 to 6 years old. Preschoolers with SLI however scored significantly lower at phonological measures at each age. Young children with SLI thus show a phonological WM delay, with a similar developmental pattern as preschoolers with normal language. WM problems in preschoolers with SLI were supported by studies of Vugs et al. (2014, 2015), in which young children with SLI were found to perform significantly below the scores of a group of typically developing children on all WM components, including not only verbal storage and verbal central executive, but also visuo-spatial storage and visuo-spatial central executive. Reduced performance on verbal WM (word span) was also found in a study in which preschool children with SLI were longitudinally compared with typically developing preschoolers (Hick et al., 2005). On the visuo-spatial WM task (pattern recall) preschoolers with SLI and typically developing children generally showed similar levels of performance over time, however, some of the SLI children scored lower on the visuo-spatial WM task and showed little improvement over time. Some children with SLI thus seem to have difficulties in the non-verbal domain of WM. The hypothesis that WM impairments in preschoolers with SLI are not restricted to verbal information but extend to non-verbal information is also supported by some studies using different visuo-spatial WM tasks (Bavin et al., 2005; Menezes et al., 2007; Marton, 2008).

WM impairment in preschoolers with SLI is supported by both parent- and teacher ratings using the Behavior Rating Inventory of Executive Function-Preschoolers (BRIEF-P: Gioia et al., 2000). That is, in a study by Wittke et al. (2013), preschoolers with SLI were rated worse on the Emergent Metacognition Index of the BRIEF-P, an index combining WM (ability of holding information in mind for the purpose of task completion) and planning/organizing scales. Further, in a study by Vugs et al. (2014) preschoolers with SLI were rated worse on the BRIEF-P's WM scale.

### Inhibition

Few empirical studies investigate inhibition in preschoolers with SLI. One study used performance based inhibition tasks, the other two used behavioral ratings of inhibition.

Spaulding (2010) investigated inhibition processes in preschoolers with SLI concentrating on two mechanisms of suppression: that is, resistance to distractor interference and inhibition of a pre-potent response. With respect to resistance to distractor interference, in SLI preschoolers performance appears to be more affected by the presented distractors (non-verbal auditory, linguistic and visual distractors) that were external and irrelevant to the goal of the task. This was evidenced by decreased accuracy for children with SLI on all distractor trials. Preschoolers with SLI seem to process both task relevant and irrelevant stimuli and therefore have more difficulty in filtering out irrelevant and distracting stimuli. The ability to suppress a pre-potent, conflicting response was assessed by using a stop-signal paradigm. Preschoolers with SLI showed poor inhibitory control, even after controlling for disparity in non-verbal cognition. From this it can be concluded that preschoolers with SLI, similar to school-age children with this disorder, have poor inhibition skills and have difficulty suppressing irrelevant information compared to their typically developing peers.

Impaired inhibition in preschoolers with SLI is supported at a behavioral level using parent- and teacher-reports on the BRIEF-P (Vugs et al., 2014) indicating that preschoolers with SLI are perceived as being less able to inhibit behavior. In contrast, Wittke et al. (2013) did not find a significant group difference between preschoolers with and without SLI on the Inhibitory Self-Control Index (combining inhibition and emotional control scales) of the BRIEF-P.

### Shifting and cognitive flexibility

Shifting or the ability to switch focus of attention between tasks or mental sets is until now investigated with a number of empirical studies using performance based tasks and/or behavioral ratings.

In a study on problem solving, a flexible item selection task was used, in which preschoolers with SLI first were asked to select two pictures with similar features, and then were asked to choose two pictures with different features (Roello et al., 2015). It was found that preschoolers with SLI show impaired cognitive flexibility compared to a group of typically developing peers. Although cognitive flexibility improved during the preschool period in SLI, the performance gap on cognitive flexibility persisted. Moreover, in another study, cognitive inflexibility was shown in preschoolers with SLI using a sorting task in which children are asked to sort cards according to switching dimensions (Farrant and Maybery, 2012). Interestingly, this cognitive inflexibility is proposed to underlie delayed Theory of Mind development in SLI.

In line with these performance based measures, Wittke et al. (2013) show that preschoolers with SLI are rated worse on the BRIEF-P's Flexibility Index which includes the scales of emotional control and shift (indexing the ability to move from one activity to another and to solve problems flexibly). Vugs et al. (2014) also find parents and teachers to rate preschoolers with SLI to perform worse on the ability to shift using the BRIEF-P. In this study a significant correlation was observed between the BRIEF-P scale of shifting and verbal storage performance.

## Discussion

From the above, it can be concluded that similar to schoolchildren with SLI, preschoolers with SLI show impairments on the three key components of EF within Miyake's model. Preschoolers with SLI show difficulties in WM, inhibition, and shifting as revealed by both performance based measures and behavioral ratings^1^.

Two broad and competing types of cognitive explanations for SLI have been put forward. Linguistic-based theories propose that SLI reflects a deficit in linguistic functioning, that is, that impairments are isolated to the language system, specifically to grammar (e.g., Rice and Wexler, 1995; Van der Lely, 2005; Stavrakaki, 2009; Rothweiler et al., 2012). In contrast, cognitive-based theories state that SLI is related to impairments in more general cognitive functioning (Gallinat and Spaulding, 2014). Herewith, those theories aim to account for the finding that children with SLI show difficulties both in linguistic and nonlinguistic domains. Some propose that the linguistic and non-linguistic impairments in SLI stem from a deficit in specific cognitive functions. For instance, limitations in verbal WM (Leonard et al., 2007) and visuo-spatial WM (Hoffman and Gillam, 2004) have both been associated with SLI. Ullman and Pierpont (2005) have come up with the procedural deficit hypothesis, linking SLI to impairments in the procedural memory system. Merzenich et al. (1993) propose impaired temporal processing to underlie symptoms of SLI. In addition to specific cognitive deficits, more general cognitive mechanisms have been proposed to underlie SLI. For instance, Bishop (1994) has put forward the limited processing account, stating that slowed processing in a system with limited processing capacity leads to linguistic errors. In all, cognitive-based theories state that an interactionist approach to cognitive functioning is needed to account for the symptomatology of SLI. Taking an interactionist perspective contributes to understanding comorbidity between developmental disorders. That is, several developmental disorders are associated with impairments in EF (like ADHD, Tannock, 1998 and ASD, Pennington and Ozonoff, 1996). Commonality of poor EF skills across developmental disorders leads to overlap in behavioral symptoms which could explain comorbidity between the classification of SLI and other developmental disorders. The executive perspective could also support differentiating developmental disorders; SLI can for example be distinguished at group level by working memory deficits which appear not specific to ADHD (Jonsdottir et al., 2005; Hutchinson et al., 2011, but see also: Alloway et al., 2009). Future research needs to point out which EF deficits are associated with ADHD, ASD and SLI exclusively and which are associated with all disorders.

Given the early onset of both EF deficits and language impairments in SLI, it is plausible that language and EF impairments interact from early childhood on. The presented results do not reveal whether executive impairments cause language impairments or vice versa. Likely, a complex and reciprocal relationship exists between EF and language. Bishop et al. (2013) propose three possible causal models for the relationship between EF and language deficits. According to the first model EF affects language processing. WM deficits, for instance, could constrain vocabulary acquisition in SLI by hindering the setting up of phonological representations in the lexicon (e.g., Gathercole, 2006; Vugs et al., 2015). Further, inhibition deficits could underlie lexical access deficits and deficient vocabulary learning in SLI. After all, inhibitory control plays a role in semantic access by enhancing and inhibiting lexical entries (Mirman and Britt, 2014) and is needed to register and disregard potential links between words and things in the world (Baldwin and Moses, 2001). According to Bishop's second model, language fuels EF development. The use of self-regulatory (inner) speech is associated with cognitive flexibility (Alarcon-Rubio et al., 2014). Also, Kuhn et al. (2015) propose that early gesture use predicts language development which then supports EF development by enabling children to build different mental representations when solving problems. In SLI, a lack of inner speech might lead to inability to keep track of instructions or to reason about problems. In the third model, language and EF co-occur because they are driven by the same factors, such as delayed development of frontal lobes which impact brain regions playing a role in language and in EF. Further research is needed to investigate which model fits best the empirical data. It is important to know if, and if yes how, training of specific EFs affects language performance. The other way around, it is valuable to examine if linguistic training enhances performance on EF tasks. Moreover, longitudinal designs may be helpful to monitor specific and detailed steps in EF and language development from early childhood to adolescence.

To end with, empirical findings on EF in preschoolers with SLI have important clinical implications. Focusing on both EF and language in neuropsychological and linguistic assessment of preschoolers with suspected SLI might contribute to early detection of SLI. Yet, future research needs to bring to light diagnostic accuracy (sensitivity/specificity) of EF and language tasks in classifying SLI (see for example Gray, 2003; Gray, who show excellent sensitivity/specificity for non-word repetition as a diagnostic measure for SLI). Cognitive differentiation will also lead to tailored treatment adding training aimed at strengthening EFs to more common language interventions. Further, in school and treatment context, demands on EF should be minimized aiming to reduce the adverse influence of possible EF impairments on learning and development.

### Conflict of interest statement

The authors declare that the research was conducted in the absence of any commercial or financial relationships that could be construed as a potential conflict of interest.

## Footnotes

^1^Importantly, the group level association between EF deficits and SLI does not imply that all individuals with SLI have EF problems. Executive heterogeneity within the SLI population needs to be further investigated.
